# Editorial: Novel approaches to foster brain plasticity in neurodevelopmental and neurodegenerative disorders

**DOI:** 10.3389/fncel.2025.1770187

**Published:** 2026-02-06

**Authors:** Anna Letizia Allegra Mascaro, Laura Baroncelli, Marco Cambiaghi

**Affiliations:** 1 Neuroscience Institute, Italian National Research Council (CNR), Pisa, Italy; 2 European Laboratory for Non-Linear Spectroscopy, Sesto Fiorentino, Italy; 3 Department of Developmental Neuroscience, IRCCS Stella Maris Foundation, Pisa, Italy; 4 Department of Neuroscience, Medicine and Movement, Universita degli Studi di Verona, Verona, Italy; 5 Department of Neurology, Hackensack Meridian School of Medicine, Nutley, NJ, United States

**Keywords:** behavioral interventions, brain plasticity, circuit modulation, epigenetic regulation, organoids, translational therapeutics

Brain plasticity, the capacity of neural circuits to rearrange in response to experience, injury, or internal state, constitutes the foundation of development, learning, and recovery. For individuals with neurodevelopmental conditions and those suffering neurodegenerative disease, harnessing and enhancing plasticity offers not only symptomatic relief but the potential to modify disease trajectories.

The Research Topic “*Novel Approaches to Foster Brain Plasticity in Neurodevelopmental and Neurodegenerative Disorders*” brings together four contributions that span model systems, molecular mechanisms, lifestyle interventions, and translational therapeutics. Collectively, these studies illuminate converging strategies to promote plasticity while also identifying critical gaps that future research must address.

In the mini-review “*Human brain organoids: An innovative model for neurological disorder research and therapy*,” Li et al. synthesize advances in organoid technology, illustrating how these three-dimensional systems can recapitulate disease-relevant circuits, cell–cell interactions, and developmental timelines. By retaining human-specific developmental trajectories and cellular diversity, organoids provide a platform for mechanistic discovery, target validation, and personalized screening. The authors also outline key limitations, including the maturation state, vascularization, and inter-regional connectivity, and ethical considerations as organoid complexity increases. The clear implication for plasticity research is that organoids can be used to probe intrinsic and extrinsic drivers of experience-dependent remodeling at human cellular resolution and to test candidate interventions that promote adaptive circuit rewiring.

The review “*Broccoli for the Brain: A Review of the Neuroprotective Mechanisms of Sulforaphane*” (Bessetti and Litwa) summarizes evidence that sulforaphane, an isothiocyanate abundant in cruciferous vegetables, activates cytoprotective transcriptional programs, reduces oxidative stress and inflammation, and supports neuronal survival and synaptic function. By restoring a permissive metabolic and inflammatory environment, such compounds may lower the threshold for adaptive plastic responses following injury or disease. While preclinical data are compelling, the authors appropriately emphasize the need for rigorously designed clinical trials to define optimal dosing, timing, and target populations.

Behavioral interventions remain among the most robust drivers of plasticity across ages. In the study “*Acceleration of spontaneous visual recovery by voluntary physical exercise in adolescent amblyopic rats*” (Marco et al.), the authors demonstrate that voluntary exercise can speed recovery of visual function in a classic model of developmental sensory deprivation. This work underscores the synergy between systemic physiological states (metabolism, neurotrophic factor levels, arousal) and local, experience-dependent synaptic remodeling. Importantly, these findings emphasize the malleability of developmental windows and show that non-pharmacological interventions can reopen or extend periods of heightened plastic potential.

Plasticity is ultimately governed by cell- and circuit-specific molecular machinery. The original research paper “*Knockdown and Overexpression of Basolateral Amygdala SIRT1 via AAV Bidirectionally Alter Morphine-Induced Conditioned Place Preference Extinction in Mice*” (Hao et al.) dissects how bidirectional manipulation of SIRT1 in a defined limbic nucleus influences extinction learning for drug-context associations. By leveraging viral-vector–mediated gene modulation and behavioral paradigms, this work links a precise epigenetic regulator to the capacity to extinguish maladaptive memories, a form of plasticity with direct relevance to addiction and possibly post-traumatic stress disorder. The study provides compelling proof of principle that circuit-specific epigenetic intervention may fine-tune plasticity in a targeted and therapeutically meaningful manner.

Despite their methodological diversity, the contributions converge on several key themes:

- **Multi-level modulation of plasticity**. Effective enhancement of plasticity can be achieved by manipulating molecular signaling (e.g., SIRT1, Nrf2), altering systemic milieu (exercise, nutrition), or using advanced biological models (organoids) to guide precision interventions. These levels are complementary and likely synergistic.
- **Timing and context matter**. Interventions that successfully promote adaptive change often depend on developmental stage, disease progression, and behavioral context. Both the adolescent exercise study and organoid maturation concerns both highlight the necessity of matching intervention timing to biological windows of receptivity.
- **Circuit specificity**. Targeting proper cells and circuits (e.g., basolateral amygdala for extinction learning) reduces off-target effects and may increase efficacy. Advances in viral vectors, gene editing, and molecular targeting open the door to highly localized plasticity modulation.
- **Host environment governs outcomes**. Oxidative stress, inflammation, metabolic state, and vascular health shape whether synapses and networks can undergo adaptive change. Approaches such as sulforaphane supplementation or exercise may act primarily by optimizing this permissive environment.

## Challenges and future directions

Translating plasticity-promoting strategies into safe, effective therapies requires addressing several challenges:

1. **Bridging model systems to humans**. Organoids and animal models provide complementary insights, but predictive validity must be rigorously tested. Parallel use of organoids, *in vivo* models, and human biomarker studies will accelerate translation.
2. **Defining biomarkers of plasticity**. Objective, non-invasive, and longitudinal biomarkers (including molecular, electrophysiological, and imaging measures) are required to quantify plastic changes.
3. **Combination therapies and individualized approaches**. Given the multiplicity of mechanisms, combinatorial regimens (e.g., targeted epigenetic modulation plus behavioral training and metabolic support) may be most effective. Patient stratification based on genetic, developmental, and metabolic profiles will be essential.

In conclusion, this Research Topic outlines an optimistic and forward-looking course: brain plasticity can be strategically modulated through complementary approaches ranging from organoid-guided target discovery and circuit-specific epigenetic tuning to dietary modulation of cellular resilience and activity-dependent behavioral interventions. The most promising translational path will integrate mechanistic precision with accessible systemic interventions, supported by rigorous biomarkers to guide personalization. As the field advances, such integrative frameworks will be essential to transform plasticity research into therapies that restore function and improve quality of life across neurodevelopmental and neurodegenerative disorders.

